# Supplementary material for: Unusual Presentation of SET::NUP214-Associated Concomitant Hematological Neoplasm in a Child—Diagnostic and Treatment Struggle
Source: Int J Mol Sci. 2023 Sep 22;24(19):14451. doi: 10.3390/ijms241914451 (PMC10572181; doi:10.3390/ijms241914451)
Supplement: Supplementary file 1 [file ijms-24-14451-s001.zip › ijms-2582353-supplementary.pdf]

Supplementary Table S1. List of 514 immunodeficiency-related genes used for DNA-based targeted NGS.

Number	Gene Symbol	Gene Name	HGNC ID	Location
1	A2ML1	alpha-2-macroglobulin like 1	HGNC:23336	12p13.31
2	ABCD4	ATP binding cassette subfamily D member 4	HGNC:68	14q24.3
3	ACD	ACD shelterin complex subunit and telomerase recruitment factor	HGNC:25070	16q22.1
4	ACP5	acid phosphatase 5, tartrate resistant	HGNC:124	19p13.2
5	ACTB	actin beta	HGNC:132	7p22.1
6	ADA	adenosine deaminase	HGNC:186	20q13.12
7	ADA2	adenosine deaminase 2	HGNC:1839	22q11.1
8	ADAM17	ADAM metallopeptidase domain 17	HGNC:195	2p25.1
9	ADAMTS3	ADAM metallopeptidase with thrombospondin type 1 motif 3	HGNC:219	4q13.3
10	ADAR	adenosine deaminase RNA specific	HGNC:225	1q21.3
11	ADGRE2	adhesion G protein-coupled receptor E2	HGNC:3337	19p13.12
12	AGA	aspartylglucosaminidase	HGNC:318	4q34.3
13	AICDA	activation induced cytidine deaminase	HGNC:13203	12p13.31
14	AIRE	autoimmune regulator	HGNC:360	21q22.3
15	AK2	adenylate kinase 2	HGNC:362	1p35.1
16	ALG12	ALG12 alpha-1,6-mannosyltransferase	HGNC:19358	22q13.33
17	ALPI	alkaline phosphatase, intestinal	HGNC:437	2q37.1
18	ANGPT1	angiopoietin 1	HGNC:484	8q23.1
19	AP1S3	adaptor related protein complex 1 subunit sigma 3	HGNC:18971	2q36.1
20	AP3B1	adaptor related protein complex 3 subunit beta 1	HGNC:566	5q14.1
21	AP3D1	adaptor related protein complex 3 subunit delta 1	HGNC:568	19p13.3
22	ARHGEF1	Rho guanine nucleotide exchange factor 1	HGNC:681	19q13.2
23	ARPC1B	actin related protein 2/3 complex subunit 1B	HGNC:704	7q22.1
24	ATM	ATM serine/threonine kinase	HGNC:795	11q22.3
25	ATP6AP1	ATPase H+ transporting accessory protein 1	HGNC:868	Xq28

26	ATR	ATR serine/threonine kinase	HGNC:882	3q23
27	AURKB	aurora kinase B	HGNC:11390	17p13.1
28	B2M	beta-2-microglobulin	HGNC:914	15q21.1
29	BACH2	BTB domain and CNC homolog 2	HGNC:14078	6q15
30	BCL10	BCL10 immune signaling adaptor	HGNC:989	1p22.3
31	BCL11B	BCL11 transcription factor B	HGNC:13222	14q32.2
32	BLM	BLM RecQ like helicase	HGNC:1058	15q26.1
33	BLNK	B cell linker	HGNC:14211	10q24.1
34	BLOC1S3	biogenesis of lysosomal organelles complex 1 subunit 3	HGNC:20914	19q13.32
35	BLOC1S6	biogenesis of lysosomal organelles complex 1 subunit 6	HGNC:8549	15q21.1
36	BTK	Bruton tyrosine kinase	HGNC:1133	Xq22.1
37	C1QA	complement C1q A chain	HGNC:1241	1p36.12
38	C1QB	complement C1q B chain	HGNC:1242	1p36.12
39	C1QC	complement C1q C chain	HGNC:1245	1p36.12
40	C1R	complement C1r	HGNC:1246	12p13.31
41	C1S	complement C1s	HGNC:1247	12p13.31
42	C2	complement C2	HGNC:1248	6p21.33
43	C3	complement C3	HGNC:1318	19p13.3
44	C3AR1	complement C3a receptor 1	HGNC:1319	12p13.31
45	C5	complement C5	HGNC:1331	9q33.2
46	C6	complement C6	HGNC:1339	5p13.1
47	C7	complement C7	HGNC:1346	5p13.1
48	C8A	complement C8 alpha chain	HGNC:1352	1p32.2
49	C8B	complement C8 beta chain	HGNC:1353	1p32.2
50	C8G	complement C8 gamma chain	HGNC:1354	9q34.3
51	C9	complement C9	HGNC:1358	5p13.1
52	CA2	carbonic anhydrase 2	HGNC:1373	8q21.2
53	CARD11	caspase recruitment domain family member 11	HGNC:16393	7p22.2

54	CARD14	caspase recruitment domain family member 14	HGNC:16446	17q25.3
55	CARD9	caspase recruitment domain family member 9	HGNC:16391	9q34.3
56	CARMIL2	capping protein regulator and myosin 1 linker 2	HGNC:27089	16q22.1
57	CASP10	caspase 10	HGNC:1500	2q33.1
58	CASP8	caspase 8	HGNC:1509	2q33.1
59	CBL	Cbl proto-oncogene	HGNC:1541	11q23.3
60	CBS	cystathionine beta-synthase	HGNC:1550	21q22.3
61	CCBE1	collagen and calcium binding EGF domains 1	HGNC:29426	18q21.32
62	CD19	CD19 molecule	HGNC:1633	16p11.2
63	CD247	CD247 molecule	HGNC:1677	1q24.2
64	CD27	CD27 molecule	HGNC:11922	12p13.31
65	CD28	CD28 molecule	HGNC:1653	2q33.2
66	CD3D	CD3 delta subunit of T-cell receptor complex	HGNC:1673	11q23.3
67	CD3E	CD3 epsilon subunit of T-cell receptor complex	HGNC:1674	11q23.3
68	CD3G	CD3 gamma subunit of T-cell receptor complex	HGNC:1675	11q23.3
69	CD40	CD40 molecule	HGNC:11919	20q13.12
70	CD40LG	CD40 ligand	HGNC:11935	Xq26.3
71	CD48	CD48 molecule	HGNC:1683	1q23.3
72	CD55	CD55 molecule (Cromer blood group)	HGNC:2665	1q32.2
73	CD59	CD59 molecule (CD59 blood group)	HGNC:1689	11p13
74	CD70	CD70 molecule	HGNC:11937	19p13.3
75	CD79A	CD79a molecule	HGNC:1698	19q13.2
76	CD79B	CD79b molecule	HGNC:1699	17q23.3
77	CD80	CD80 molecule	HGNC:1700	3q13.33
78	CD81	CD81 molecule	HGNC:1701	11p15.5
79	CD8A	CD8 subunit alpha	HGNC:1706	2p11.2
80	CDC42	cell division cycle 42	HGNC:1736	1p36.12
81	CDCA7	cell division cycle associated 7	HGNC:14628	2q31.1

82	CEBPE	CCAAT enhancer binding protein epsilon	HGNC:1836	14q11.2
83	CFB	complement factor B	HGNC:1037	6p21.33
84	CFD	complement factor D	HGNC:2771	19p13.3
85	CFH	complement factor H	HGNC:4883	1q31.3
86	CFI	complement factor I	HGNC:5394	4q25
87	CFP	complement factor properdin	HGNC:8864	Xp11.23
88	CFTR	CF transmembrane conductance regulator	HGNC:1884	7q31.2
89	CHD7	chromodomain helicase DNA binding protein 7	HGNC:20626	8q12.2
90	CIB1	calcium and integrin binding 1	HGNC:16920	15q26.1
91	CIITA	class II major histocompatibility complex transactivator	HGNC:7067	16p13.13
92	CLCN7	chloride voltage-gated channel 7	HGNC:2025	16p13.3
93	CLPB	caseinolytic mitochondrial matrix peptidase chaperone subunit B	HGNC:30664	11q13.4
94	COG6	component of oligomeric golgi complex 6	HGNC:18621	13q14.11
95	COL2A1	collagen type II alpha 1 chain	HGNC:2200	12q13.11
96	COL7A1	collagen type VII alpha 1 chain	HGNC:2214	3p21.31
97	COPA	COPI coat complex subunit alpha	HGNC:2230	1q23.2
98	COPG1	COPI coat complex subunit gamma 1	HGNC:2236	3q21.3
99	COPZ1	COPI coat complex subunit zeta 1	HGNC:2243	12q13.13
100	CORO1A	coronin 1A	HGNC:2252	16p11.2
101	CR2	complement C3d receptor 2	HGNC:2336	1q32.2
102	CSF2RA	colony stimulating factor 2 receptor subunit alpha	HGNC:2435	Xp22.32 and Yp11.3
103	CSF2RB	colony stimulating factor 2 receptor subunit beta	HGNC:2436	22q12.3
104	CSF3R	colony stimulating factor 3 receptor	HGNC:2439	1p34.3
105	CTC1	CST telomere replication complex component 1	HGNC:26169	17p13.1
106	CTLA4	cytotoxic T-lymphocyte associated protein 4	HGNC:2505	2q33.2
107	CTPS1	CTP synthase 1	HGNC:2519	1p34.2
108	CTSC	cathepsin C	HGNC:2528	11q14.2
109	CXCR4	C-X-C motif chemokine receptor 4	HGNC:2561	2q22.1

110	CYBA	cytochrome b-245 alpha chain	HGNC:2577	16q24.2
111	CYBB	cytochrome b-245 beta chain	HGNC:2578	Xp21.1-p11.4
112	CYBC1	cytochrome b-245 chaperone 1	HGNC:28672	17q25.3
113	DBF4	DBF4 zinc finger	HGNC:17364	7q21.12
114	DBR1	debranching RNA lariats 1	HGNC:15594	3q22.3
115	DCLRE1C	DNA cross-link repair 1C	HGNC:17642	10p13
116	RIGI	RNA sensor RIG-I	HGNC:19102	9p21.1
117	DEF6	DEF6 guanine nucleotide exchange factor	HGNC:2760	6p21.31
118	DIAPH1	diaphanous related formin 1	HGNC:2876	5q31.3
119	DKC1	dyskerin pseudouridine synthase 1	HGNC:2890	Xq28
120	DNASE1	deoxyribonuclease 1	HGNC:2956	16p13.3
121	DNASE1L3	deoxyribonuclease 1 like 3	HGNC:2959	3p14.3
122	DNASE2	deoxyribonuclease 2, lysosomal	HGNC:2960	19p13.13
123	DNMT3B	DNA methyltransferase 3 beta	HGNC:2979	20q11.21
124	DOCK11	dedicator of cytokinesis 11	HGNC:23483	Xq24
125	DOCK2	dedicator of cytokinesis 2	HGNC:2988	5q35.1
126	DOCK8	dedicator of cytokinesis 8	HGNC:19191	9p24.3
127	DPAGT1	dolichyl-phosphate N-acetylglucosaminephosphotransferase 1	HGNC:2995	11q23.3
128	DSG1	desmoglein 1	HGNC:3048	18q12.1
129	DTNBP1	dystrobrevin binding protein 1	HGNC:17328	6p22.3
130	EDA	ectodysplasin A	HGNC:3157	Xq13.1
131	EDAR	ectodysplasin A receptor	HGNC:2895	2q13
132	EDARADD	EDAR associated via death domain	HGNC:14341	1q42.3-q43
133	EFL1	elongation factor like GTPase 1	HGNC:25789	15q25.2
134	EGFR	epidermal growth factor receptor	HGNC:3236	7p11.2
135	EIF6	eukaryotic translation initiation factor 6	HGNC:6159	20q11.22
136	ELANE	elastase, neutrophil expressed	HGNC:3309	19p13.3
137	ELP1	elongator acetyltransferase complex subunit 1	HGNC:5959	9q31.3

138	EP300	E1A binding protein p300	HGNC:3373	22q13.2
139	EPCAM	epithelial cell adhesion molecule	HGNC:11529	2p21
140	EPG5	ectopic P-granules 5 autophagy tethering factor	HGNC:29331	18q12.3-q21.1
141	ERBIN	erbb2 interacting protein	HGNC:15842	5q12.3
142	EXTL3	exostosin like glycosyltransferase 3	HGNC:3518	8p21.1
143	F12	coagulation factor XII	HGNC:3530	5q35.3
144	FADD	Fas associated via death domain	HGNC:3573	11q13.3
145	FAM111B	FAM111 trypsin like peptidase B	HGNC:24200	11q12.1
146	FAS	Fas cell surface death receptor	HGNC:11920	10q23.31
147	FASLG	Fas ligand	HGNC:11936	1q24.3
148	FAT4	FAT atypical cadherin 4	HGNC:23109	4q28.1
149	FBN1	fibrillin 1	HGNC:3603	15q21.1
150	FCGR3A	Fc gamma receptor IIIa	HGNC:3619	1q23.3
151	FCGR3B	Fc gamma receptor IIIb	HGNC:3620	1q23.3
152	FCHO1	FCH and mu domain containing endocytic adaptor 1	HGNC:29002	19p13.11
153	FECH	ferrochelatase	HGNC:3647	18q21.31
154	FERMT1	FERM domain containing kindlin 1	HGNC:15889	20p12.3
155	FERMT3	FERM domain containing kindlin 3	HGNC:23151	11q13.1
156	FNIP1	folliculin interacting protein 1	HGNC:29418	5q31.1
157	FOXN1	forkhead box N1	HGNC:12765	17q11.2
158	FOXP3	forkhead box P3	HGNC:6106	Xp11.23
159	G6PC3	glucose-6-phosphatase catalytic subunit 3	HGNC:24861	17q21.31
160	GATA1	GATA binding protein 1	HGNC:4170	Xp11.23
161	GATA2	GATA binding protein 2	HGNC:4171	3q21.3
162	GBA1	glucosylceramidase beta 1	HGNC:4177	1q22
163	GFI1	growth factor independent 1 transcriptional repressor	HGNC:4237	1p22.1
164	GIMAP6	GTPase, IMAP family member 6	HGNC:21918	7q36.1
165	GINS1	GINS complex subunit 1	HGNC:28980	20p11.21

166	GJC2	gap junction protein gamma 2	HGNC:17494	1q42.13
167	GLA	galactosidase alpha	HGNC:4296	Xq22.1
168	GUCY2C	guanylate cyclase 2C	HGNC:4688	12p12.3
169	HAVCR2	hepatitis A virus cellular receptor 2	HGNC:18437	5q33.3
170	HAX1	HCLS1 associated protein X-1	HGNC:16915	1q21.3
171	HELLS	helicase, lymphoid specific	HGNC:4861	10q23.33
172	HFE	homeostatic iron regulator	HGNC:4886	6p22.2
173	HMOX1	heme oxygenase 1	HGNC:5013	22q12.3
174	HPS1	HPS1 biogenesis of lysosomal organelles complex 3 subunit 1	HGNC:5163	10q24.2
175	HPS3	HPS3 biogenesis of lysosomal organelles complex 2 subunit 1	HGNC:15597	3q24
176	HPS4	HPS4 biogenesis of lysosomal organelles complex 3 subunit 2	HGNC:15844	22q12.1
177	HPS5	HPS5 biogenesis of lysosomal organelles complex 2 subunit 2	HGNC:17022	11p15.1
178	HPS6	HPS6 biogenesis of lysosomal organelles complex 2 subunit 3	HGNC:18817	10q24.32
179	HRAS	HRas proto-oncogene, GTPase	HGNC:5173	11p15.5
180	HSPA1L	heat shock protein family A (Hsp70) member 1 like	HGNC:5234	6p21.33
181	HYOU1	hypoxia up-regulated 1	HGNC:16931	11q23.3
182	ICOS	inducible T cell costimulator	HGNC:5351	2q33.2
183	ICOSLG	inducible T cell costimulator ligand	HGNC:17087	21q22.3
184	IFIH1	interferon induced with helicase C domain 1	HGNC:18873	2q24.2
185	IFNAR1	interferon alpha and beta receptor subunit 1	HGNC:5432	21q22.11
186	IFNAR2	interferon alpha and beta receptor subunit 2	HGNC:5433	21q22.11
187	IFNG	interferon gamma	HGNC:5438	12q15
188	IFNGR1	interferon gamma receptor 1	HGNC:5439	6q23.3
189	IFNGR2	interferon gamma receptor 2	HGNC:5440	21q22.11
190	IGHM	immunoglobulin heavy constant mu	HGNC:5541	14q32.33
191	IGKC	immunoglobulin kappa constant	HGNC:5716	2p11.2
192	IGLL1	immunoglobulin lambda like polypeptide 1	HGNC:5870	22q11.23
193	IKBKB	inhibitor of nuclear factor kappa B kinase subunit beta	HGNC:5960	8p11.21

194	IKBKG	inhibitor of nuclear factor kappa B kinase regulatory subunit gamma	HGNC:5961	Xq28
195	IKZF1	IKAROS family zinc finger 1	HGNC:13176	7p12.2
196	IL10	interleukin 10	HGNC:5962	1q32.1
197	IL10RA	interleukin 10 receptor subunit alpha	HGNC:5964	11q23.3
198	IL10RB	interleukin 10 receptor subunit beta	HGNC:5965	21q22.11
199	IL11RA	interleukin 11 receptor subunit alpha	HGNC:5967	9p13.3
200	IL12B	interleukin 12B	HGNC:5970	5q33.3
201	IL12RB1	interleukin 12 receptor subunit beta 1	HGNC:5971	19p13.11
202	IL12RB2	interleukin 12 receptor subunit beta 2	HGNC:5972	1p31.3
203	IL17F	interleukin 17F	HGNC:16404	6p12.2
204	IL17RA	interleukin 17 receptor A	HGNC:5985	22q11.1
205	IL17RC	interleukin 17 receptor C	HGNC:18358	3p25.3
206	IL18BP	interleukin 18 binding protein	HGNC:5987	11q13.4
207	IL1RN	interleukin 1 receptor antagonist	HGNC:6000	2q14.1
208	IL21	interleukin 21	HGNC:6005	4q27
209	IL21R	interleukin 21 receptor	HGNC:6006	16p12.1
210	IL2RA	interleukin 2 receptor subunit alpha	HGNC:6008	10p15.1
211	IL2RB	interleukin 2 receptor subunit beta	HGNC:6009	22q12.3
212	IL2RG	interleukin 2 receptor subunit gamma	HGNC:6010	Xq13.1
213	IL36RN	interleukin 36 receptor antagonist	HGNC:15561	2q14.1
214	IL6R	interleukin 6 receptor	HGNC:6019	1q21.3
215	IL6ST	interleukin 6 cytokine family signal transducer	HGNC:6021	5q11.2
216	IL7R	interleukin 7 receptor	HGNC:6024	5p13.2
217	IRAK4	interleukin 1 receptor associated kinase 4	HGNC:17967	12q12
218	IRF2BP2	interferon regulatory factor 2 binding protein 2	HGNC:21729	1q42.3
219	IRF3	interferon regulatory factor 3	HGNC:6118	19q13.33
220	IRF4	interferon regulatory factor 4	HGNC:6119	6p25.3
221	IRF7	interferon regulatory factor 7	HGNC:6122	11p15.5

222	IRF8	interferon regulatory factor 8	HGNC:5358	16q24.1
223	IRF9	interferon regulatory factor 9	HGNC:6131	14q12
224	ISG15	ISG15 ubiquitin like modifier	HGNC:4053	1p36.33
225	ITCH	itchy E3 ubiquitin protein ligase	HGNC:13890	20q11.22
226	ITGA3	integrin subunit alpha 3	HGNC:6139	17q21.33
227	ITGB2	integrin subunit beta 2	HGNC:6155	21q22.3
228	ITGB4	integrin subunit beta 4	HGNC:6158	17q25.1
229	ITK	IL2 inducible T cell kinase	HGNC:6171	5q33.3
230	ITPKB	inositol-trisphosphate 3-kinase B	HGNC:6179	1q42.12
231	ITPR3	inositol 1,4,5-trisphosphate receptor type 3	HGNC:6182	6p21.31
232	IVD	isovaleryl-CoA dehydrogenase	HGNC:6186	15q15.1
233	IVNS1ABP	influenza virus NS1A binding protein	HGNC:16951	1q25.3
234	JAGN1	jagunal homolog 1	HGNC:26926	3p25.3
235	JAK1	Janus kinase 1	HGNC:6190	1p31.3
236	JAK3	Janus kinase 3	HGNC:6193	19p13.11
237	KDM1A	lysine demethylase 1A	HGNC:29079	1p36.12
238	KDM6A	lysine demethylase 6A	HGNC:12637	Xp11.3
239	KMT2A	lysine methyltransferase 2A	HGNC:7132	11q23.3
240	KMT2D	lysine methyltransferase 2D	HGNC:7133	12q13.12
241	KNG1	kininogen 1	HGNC:6383	3q27.3
242	KRAS	KRAS proto-oncogene, GTPase	HGNC:6407	12p12.1
243	LACC1	laccase domain containing 1	HGNC:26789	13q14.11
244	LAMA3	laminin subunit alpha 3	HGNC:6483	18q11.2
245	LAMB3	laminin subunit beta 3	HGNC:6490	1q32.2
246	LAMC2	laminin subunit gamma 2	HGNC:6493	1q25.3
247	LAMTOR2	late endosomal/lysosomal adaptor, MAPK and MTOR activator 2	HGNC:29796	1q22
248	LAT	linker for activation of T cells	HGNC:18874	16q13
249	LCK	LCK proto-oncogene, Src family tyrosine kinase	HGNC:6524	1p35.2

250	LCP2	lymphocyte cytosolic protein 2	HGNC:6529	5q35.1
251	LIG1	DNA ligase 1	HGNC:6598	19q13.33
252	LIG4	DNA ligase 4	HGNC:6601	13q33.3
253	LMBRD1	LMBR1 domain containing 1	HGNC:23038	6q13
254	LPIN2	lipin 2	HGNC:14450	18p11.31
255	LRBA	LPS responsive beige-like anchor protein	HGNC:1742	4q31.3
256	LRP5	LDL receptor related protein 5	HGNC:6697	11q13.2
257	LRRC8A	leucine rich repeat containing 8 VRAC subunit A	HGNC:19027	9q34.11
258	LYN	LYN proto-oncogene, Src family tyrosine kinase	HGNC:6735	8q12.1
259	LYST	lysosomal trafficking regulator	HGNC:1968	1q42.3
260	LZTR1	leucine zipper like transcription regulator 1	HGNC:6742	22q11.21
261	MAGT1	magnesium transporter 1	HGNC:28880	Xq21.1
262	MALT1	MALT1 paracaspase	HGNC:6819	18q21.32
263	MAN2B1	mannosidase alpha class 2B member 1	HGNC:6826	19p13.13
264	MAP2K1	mitogen-activated protein kinase kinase 1	HGNC:6840	15q22.31
265	MAP2K2	mitogen-activated protein kinase kinase 2	HGNC:6842	19p13.3
266	MAP3K14	mitogen-activated protein kinase kinase kinase 14	HGNC:6853	17q21.31
267	MCM4	minichromosome maintenance complex component 4	HGNC:6947	8q11.21
268	MEFV	MEFV innate immunity regulator, pyrin	HGNC:6998	16p13.3
269	MMAA	metabolism of cobalamin associated A	HGNC:18871	4q31.21
270	MMAB	metabolism of cobalamin associated B	HGNC:19331	12q24.11
271	MMACHC	metabolism of cobalamin associated C	HGNC:24525	1p34.1
272	MMADHC	metabolism of cobalamin associated D	HGNC:25221	2q23.2
273	MMUT	methylmalonyl-CoA mutase	HGNC:7526	6p12.3
274	MOGS	mannosyl-oligosaccharide glucosidase	HGNC:24862	2p13.1
275	MPO	myeloperoxidase	HGNC:7218	17q22
276	MRE11	MRE11 homolog, double strand break repair nuclease	HGNC:7230	11q21
277	MRTFA	myocardin related transcription factor A	HGNC:14334	22q13.1-q13.2

278	MS4A1	membrane spanning 4-domains A1	HGNC:7315	11q12.2
279	MSN	moesin	HGNC:7373	Xq12
280	MTHFD1	methylenetetrahydrofolate dehydrogenase, cyclohydrolase and formyltetrahydrofolate synthetase 1	HGNC:7432	14q23.3
281	MTRR	5-methyltetrahydrofolate-homocysteine methyltransferase reductase	HGNC:7473	5p15.31
282	MVK	mevalonate kinase	HGNC:7530	12q24.11
283	MYD88	MYD88 innate immune signal transduction adaptor	HGNC:7562	3p22.2
284	MYO5A	myosin VA	HGNC:7602	15q21.2
285	MYO5B	myosin VB	HGNC:7603	18q
286	MYOF	myoferlin	HGNC:3656	10q23.33
287	MYSM1	Myb like, SWIRM and MPN domains 1	HGNC:29401	1p32.1
288	NBAS	NBAS subunit of NRZ tethering complex	HGNC:15625	2p24.3
289	NBN	nibrin	HGNC:7652	8q21.3
290	NCF1	neutrophil cytosolic factor 1	HGNC:7660	7q11.23
291	NCF2	neutrophil cytosolic factor 2	HGNC:7661	1q25.3
292	NCF4	neutrophil cytosolic factor 4	HGNC:7662	22q12.3
293	NCKAP1L	NCK associated protein 1 like	HGNC:4862	12q13.13-q13.2
294	NCSTN	nicastrin	HGNC:17091	1q23.2
295	NF1	neurofibromin 1	HGNC:7765	17q11.2
296	NF2	NF2, moesin-ezrin-radixin like (MERLIN) tumor suppressor	HGNC:7773	22q12.2
297	NFE2L2	NFE2 like bZIP transcription factor 2	HGNC:7782	2q31.2
298	NFIL3	nuclear factor, interleukin 3 regulated	HGNC:7787	9q22.31
299	NFKB1	nuclear factor kappa B subunit 1	HGNC:7794	4q24
300	NFKB2	nuclear factor kappa B subunit 2	HGNC:7795	10q24.32
301	NFKBIA	NFKB inhibitor alpha	HGNC:7797	14q13.2
302	NHEJ1	non-homologous end joining factor 1	HGNC:25737	2q35
303	NHP2	NHP2 ribonucleoprotein	HGNC:14377	5q35.3
304	NLRC4	NLR family CARD domain containing 4	HGNC:16412	2p22.3
305	NLRP1	NLR family pyrin domain containing 1	HGNC:14374	17p13

306	NLRP12	NLR family pyrin domain containing 12	HGNC:22938	19q13.42
307	NLRP3	NLR family pyrin domain containing 3	HGNC:16400	1q44
308	NLRP6	NLR family pyrin domain containing 6	HGNC:22944	11p15.5
309	NOD2	nucleotide binding oligomerization domain containing 2	HGNC:5331	16q12.1
310	NOP10	NOP10 ribonucleoprotein	HGNC:14378	15q14
311	NOS2	nitric oxide synthase 2	HGNC:7873	17q11.2
312	NRAS	NRAS proto-oncogene, GTPase	HGNC:7989	1p13.2
313	NSMCE3	NSE3 homolog, SMC5-SMC6 complex component	HGNC:7677	15q13.1
314	NUP214	nucleoporin 214	HGNC:8064	9q34.13
315	OAS1	2'-5'-oligoadenylate synthetase 1	HGNC:8086	12q24.13
316	ORAI1	ORAI calcium release-activated calcium modulator 1	HGNC:25896	12q24.31
317	OSTM1	osteoclastogenesis associated transmembrane protein 1	HGNC:21652	6q21
318	OTULIN	OTU deubiquitinase with linear linkage specificity	HGNC:25118	5p15.2
319	PARN	poly(A)-specific ribonuclease	HGNC:8609	16p13.12
320	PCCA	propionyl-CoA carboxylase subunit alpha	HGNC:8653	13q32.3
321	PCCB	propionyl-CoA carboxylase subunit beta	HGNC:8654	3q22.3
322	PEPD	peptidase D	HGNC:8840	19q13.11
323	PGM3	phosphoglucomutase 3	HGNC:8907	6q14.1
324	PIK3CD	phosphatidylinositol-4,5-bisphosphate 3-kinase catalytic subunit delta	HGNC:8977	1p36.22
325	PIK3CG	phosphatidylinositol-4,5-bisphosphate 3-kinase catalytic subunit gamma	HGNC:8978	7q22.3
326	PIK3R1	phosphoinositide-3-kinase regulatory subunit 1	HGNC:8979	5q13.1
327	PLCG2	phospholipase C gamma 2	HGNC:9066	16q24.1
328	PLEKHM1	pleckstrin homology and RUN domain containing M1	HGNC:29017	17q21.31
329	PLG	plasminogen	HGNC:9071	6q26
330	PLVAP	plasmalemma vesicle associated protein	HGNC:13635	19p13.11
331	PMM2	phosphomannomutase 2	HGNC:9115	16p13.2
332	PNP	purine nucleoside phosphorylase	HGNC:7892	14q11.2
333	POGLUT1	protein O-glucosyltransferase 1	HGNC:22954	3q13.33

334	POLA1	DNA polymerase alpha 1, catalytic subunit	HGNC:9173	Xp22.11-p21.3
335	POLD1	DNA polymerase delta 1, catalytic subunit	HGNC:9175	19q13.3
336	POLD2	DNA polymerase delta 2, accessory subunit	HGNC:9176	7p13
337	POLD3	DNA polymerase delta 3, accessory subunit	HGNC:20932	11q13.4
338	POLE	DNA polymerase epsilon, catalytic subunit	HGNC:9177	12q24.33
339	POLE2	DNA polymerase epsilon 2, accessory subunit	HGNC:9178	14q21.3
340	POLR3A	RNA polymerase III subunit A	HGNC:30074	10q22.3
341	POLR3C	RNA polymerase III subunit C	HGNC:30076	1q21.1
342	POLR3F	RNA polymerase III subunit F	HGNC:15763	20p11.23
343	POMP	proteasome maturation protein	HGNC:20330	13q12.3
344	POU2AF1	POU class 2 homeobox associating factor 1	HGNC:9211	11q23.1
345	PRDX1	peroxiredoxin 1	HGNC:9352	1p34.1
346	PRF1	perforin 1	HGNC:9360	10q22.1
347	PRKCD	protein kinase C delta	HGNC:9399	3p21.1
348	PRKDC	protein kinase, DNA-activated, catalytic subunit	HGNC:9413	8q11.21
349	PSEN1	presenilin 1	HGNC:9508	14q24.2
350	PSENEN	presenilin enhancer, gamma-secretase subunit	HGNC:30100	19q13.12
351	PSMA3	proteasome 20S subunit alpha 3	HGNC:9532	14q23.1
352	PSMA5	proteasome 20S subunit alpha 5	HGNC:9534	1p13.3
353	PSMB10	proteasome 20S subunit beta 10	HGNC:9538	16q22.1
354	PSMB4	proteasome 20S subunit beta 4	HGNC:9541	1q21.3
355	PSMB8	proteasome 20S subunit beta 8	HGNC:9545	6p21.32
356	PSMB9	proteasome 20S subunit beta 9	HGNC:9546	6p21.32
357	PSMC5	proteasome 26S subunit, ATPase 5	HGNC:9552	17q23.3
358	PSMG2	proteasome assembly chaperone 2	HGNC:24929	18p11.21
359	PSTPIP1	proline-serine-threonine phosphatase interacting protein 1	HGNC:9580	15q24.3
360	PTCRA	pre T cell antigen receptor alpha	HGNC:21290	6p21.1
361	PTEN	phosphatase and tensin homolog	HGNC:9588	10q23.31

362	PTPN11	protein tyrosine phosphatase non-receptor type 11	HGNC:9644	12q24.13
363	PTPN2	protein tyrosine phosphatase non-receptor type 2	HGNC:9650	18p11.21
364	PTPRC	protein tyrosine phosphatase receptor type C	HGNC:9666	1q31.3-q32.1
365	RAB27A	RAB27A, member RAS oncogene family	HGNC:9766	15q21.3
366	RAC2	Rac family small GTPase 2	HGNC:9802	22q13.1
367	RAG1	recombination activating 1	HGNC:9831	11p12
368	RAG2	recombination activating 2	HGNC:9832	11p12
369	RANBP2	RAN binding protein 2	HGNC:9848	2q13
370	RASA2	RAS p21 protein activator 2	HGNC:9872	3q23
371	RASGRP1	RAS guanyl releasing protein 1	HGNC:9878	15q14
372	RBCK1	RANBP2-type and C3HC4-type zinc finger containing 1	HGNC:15864	20p13
373	RC3H1	ring finger and CCCH-type domains 1	HGNC:29434	1q25.1
374	REL	REL proto-oncogene, NF-kB subunit	HGNC:9954	2p16.1
375	RELA	RELA proto-oncogene, NF-kB subunit	HGNC:9955	11q13.1
376	RELB	RELB proto-oncogene, NF-kB subunit	HGNC:9956	19q13.32
377	RFX5	regulatory factor X5	HGNC:9986	1q21.3
378	RFXANK	regulatory factor X associated ankyrin containing protein	HGNC:9987	19p13.11
379	RFXAP	regulatory factor X associated protein	HGNC:9988	13q13.3
380	RHOG	ras homolog family member G	HGNC:672	11p15.4
381	RHOH	ras homolog family member H	HGNC:686	4p14
382	RIPK1	receptor interacting serine/threonine kinase 1	HGNC:10019	6p25.2
383	RMRP	RNA component of mitochondrial RNA processing endoribonuclease	HGNC:10031	9p13.3
384	RNASEH2A	ribonuclease H2 subunit A	HGNC:18518	19p13.13
385	RNASEH2B	ribonuclease H2 subunit B	HGNC:25671	13q14.3
386	RNASEH2C	ribonuclease H2 subunit C	HGNC:24116	11q13.1
387	RNF168	ring finger protein 168	HGNC:26661	3q29
388	RNF31	ring finger protein 31	HGNC:16031	14q12
389	RNU4ATAC	RNA, U4atac small nuclear	HGNC:34016	2q14.2

390	RORC	RAR related orphan receptor C	HGNC:10260	1q21
391	RPSA	ribosomal protein SA	HGNC:6502	3p22.1
392	RRAS	RAS related	HGNC:10447	19q13.33
393	RRAS2	RAS related 2	HGNC:17271	11p15.2
394	RTEL1	regulator of telomere elongation helicase 1	HGNC:15888	20q13.33
395	SAMD9L	sterile alpha motif domain containing 9 like	HGNC:1349	7q21.2
396	SAMHD1	SAM and HD domain containing deoxynucleoside triphosphate triphosphohydrolase 1	HGNC:15925	20q11.23
397	SASH3	SAM and SH3 domain containing 3	HGNC:15975	Xq26.1
398	SBDS	SBDS ribosome maturation factor	HGNC:19440	7q11.21
399	SC5D	sterol-C5-desaturase	HGNC:10547	11q23.3-q24.1
400	SEC61A1	SEC61 translocon subunit alpha 1	HGNC:18276	3q21.3
401	SEMA3E	semaphorin 3E	HGNC:10727	7q21.11
402	SERPINB1	serpin family B member 1	HGNC:3311	6p25.2
403	SERPING1	serpin family G member 1	HGNC:1228	11q12.1
404	SH2D1A	SH2 domain containing 1A	HGNC:10820	Xq25
405	SH3BP2	SH3 domain binding protein 2	HGNC:10825	4p16.3
406	SH3KBP1	SH3 domain containing kinase binding protein 1	HGNC:13867	Xp22.12
407	SHARPIN	SHANK associated RH domain interactor	HGNC:25321	8q24.3
408	SKIC2	SKI2 subunit of superkiller complex	HGNC:10898	6p21.33
409	SLC29A3	solute carrier family 29 member 3	HGNC:23096	10q22.1
410	SLC35A1	solute carrier family 35 member A1	HGNC:11021	6q15
411	SLC35C1	solute carrier family 35 member C1	HGNC:20197	11p11.2
412	SLC37A4	solute carrier family 37 member 4	HGNC:4061	11q23.3
413	SLC39A4	solute carrier family 39 member 4	HGNC:17129	8q24.3
414	SLC39A7	solute carrier family 39 member 7	HGNC:4927	6p21.32
415	SLC39A8	solute carrier family 39 member 8	HGNC:20862	4q24
416	SLC46A1	solute carrier family 46 member 1	HGNC:30521	17q11.2
417	SLC7A7	solute carrier family 7 member 7	HGNC:11065	14q11.2

418	SLCO2A1	solute carrier organic anion transporter family member 2A1	HGNC:10955	3q22.1-q22.2
419	SMARCAL1	SWI/SNF related, matrix associated, actin dependent regulator of chromatin, subfamily a like 1	HGNC:11102	2q35
420	SMARCD2	SWI/SNF related, matrix associated, actin dependent regulator of chromatin, subfamily d, member 2	HGNC:11107	17q23.3
421	SMC1A	structural maintenance of chromosomes 1A	HGNC:11111	Xp11.22
422	SMC3	structural maintenance of chromosomes 3	HGNC:2468	10q25.2
423	SMPD1	sphingomyelin phosphodiesterase 1	HGNC:11120	11p15.4
424	SNORA31	small nucleolar RNA, H/ACA box 31	HGNC:32621	13q14.13
425	SNX10	sorting nexin 10	HGNC:14974	7p15.2
426	SOCS1	suppressor of cytokine signaling 1	HGNC:19383	16p13.13
427	SP1	Sp1 transcription factor	HGNC:11205	12q13.13
428	SP110	SP110 nuclear body protein	HGNC:5401	2q37.1
429	SPI1	Spi-1 proto-oncogene	HGNC:11241	11p11.2
430	SPINK5	serine peptidase inhibitor Kazal type 5	HGNC:15464	5q32
431	SPPL2A	signal peptide peptidase like 2A	HGNC:30227	15q21.2
432	SRP54	signal recognition particle 54	HGNC:11301	14q13.2
433	STAT1	signal transducer and activator of transcription 1	HGNC:11362	2q32.2
434	STAT2	signal transducer and activator of transcription 2	HGNC:11363	12q13.3
435	STAT3	signal transducer and activator of transcription 3	HGNC:11364	17q21.2
436	STAT5B	signal transducer and activator of transcription 5B	HGNC:11367	17q21.2
437	STIM1	stromal interaction molecule 1	HGNC:11386	11p15.4
438	STING1	stimulator of interferon response cGAMP interactor 1	HGNC:27962	5q31.2
439	STK4	serine/threonine kinase 4	HGNC:11408	20q13.12
440	STN1	STN1 subunit of CST complex	HGNC:26200	10q24.33
441	STX11	syntaxin 11	HGNC:11429	6q24.2
442	STXBP2	syntaxin binding protein 2	HGNC:11445	19p13.2
443	STXBP3	syntaxin binding protein 3	HGNC:11446	1p13.3
444	TAP1	transporter 1, ATP binding cassette subfamily B member	HGNC:43	6p21.32
445	TAP2	transporter 2, ATP binding cassette subfamily B member	HGNC:44	6p21.32

446	TAPBP	TAP binding protein	HGNC:11566	6p21.32
447	TAFAZZIN	tafazzin, phospholipid-lysophospholipid transacylase	HGNC:11577	Xq28
448	TBK1	TANK binding kinase 1	HGNC:11584	12q14.2
449	TBX1	T-box transcription factor 1	HGNC:11592	22q11.21
450	TBX21	T-box transcription factor 21	HGNC:11599	17q21.32
451	TCF3	transcription factor 3	HGNC:11633	19p13.3
452	TCIRG1	T cell immune regulator 1, ATPase H+ transporting V0 subunit a3	HGNC:11647	11q13.2
453	TCN2	transcobalamin 2	HGNC:11653	22q12.2
454	TERC	telomerase RNA component	HGNC:11727	3q26.2
455	TERT	telomerase reverse transcriptase	HGNC:11730	5p15.33
456	TFR2	transferrin receptor 2	HGNC:11762	7q22.1
457	TFRC	transferrin receptor	HGNC:11763	3q29
458	TGFBR1	transforming growth factor beta receptor 1	HGNC:11772	9q22.33
459	TGFBR2	transforming growth factor beta receptor 2	HGNC:11773	3p24.1
460	THBD	thrombomodulin	HGNC:11784	20p11.21
461	TICAM1	TIR domain containing adaptor molecule 1	HGNC:18348	19p13.3
462	TIMM50	translocase of inner mitochondrial membrane 50	HGNC:23656	19q13.2
463	TINF2	TERF1 interacting nuclear factor 2	HGNC:11824	14q12
464	TLR3	toll like receptor 3	HGNC:11849	4q35.1
465	TLR7	toll like receptor 7	HGNC:15631	Xp22.2
466	TLR8	toll like receptor 8	HGNC:15632	Xp22.2
467	TMC6	transmembrane channel like 6	HGNC:18021	17q25.3
468	TMC8	transmembrane channel like 8	HGNC:20474	17q25.3
469	TNFAIP3	TNF alpha induced protein 3	HGNC:11896	6q23.3
470	TNFRSF11A	TNF receptor superfamily member 11a	HGNC:11908	18q21.33
471	TNFRSF13B	TNF receptor superfamily member 13B	HGNC:18153	17p11.2
472	TNFRSF13C	TNF receptor superfamily member 13C	HGNC:17755	22q13.2
473	TNFRSF1A	TNF receptor superfamily member 1A	HGNC:11916	12p13.31

474	TNFRSF4	TNF receptor superfamily member 4	HGNC:11918	1p36.33
475	TNFRSF9	TNF receptor superfamily member 9	HGNC:11924	1p36.23
476	TNFSF11	TNF superfamily member 11	HGNC:11926	13q14
477	TNFSF12	TNF superfamily member 12	HGNC:11927	17p13.1
478	TNFSF13	TNF superfamily member 13	HGNC:11928	17p13.1
479	TOM1	target of myb1 membrane trafficking protein	HGNC:11982	22q12.3
480	TOP2B	DNA topoisomerase II beta	HGNC:11990	3p24.2
481	TPP1	tripeptidyl peptidase 1	HGNC:2073	11p15.4
482	TPP2	tripeptidyl peptidase 2	HGNC:12016	13q33.1
483	TRAC	T cell receptor alpha constant	HGNC:12029	14q11.2
484	TRAF3	TNF receptor associated factor 3	HGNC:12033	14q32.32
485	TRAF3IP2	TRAF3 interacting protein 2	HGNC:1343	6q21
486	TREX1	three prime repair exonuclease 1	HGNC:12269	3p21.31
487	TRIM22	tripartite motif containing 22	HGNC:16379	11p15.4
488	TRNT1	tRNA nucleotidyl transferase 1	HGNC:17341	3p26.2
489	TSPEAR	thrombospondin type laminin G domain and EAR repeats	HGNC:1268	21q22.3
490	SKIC3	SKI3 subunit of superkiller complex	HGNC:23639	5q15
491	TTC7A	tetratricopeptide repeat domain 7A	HGNC:19750	2p21
492	TYK2	tyrosine kinase 2	HGNC:12440	19p13.2
493	UBA1	Ubiquitin Like Modifier Activating Enzyme 1	HGNC:12469	Xp11.3
494	UNC13D	unc-13 homolog D	HGNC:23147	17q25.3
495	UNC93B1	unc-93 homolog B1, TLR signaling regulator	HGNC:13481	11q13.2
496	UNG	uracil DNA glycosylase	HGNC:12572	12q24.11
497	USB1	U6 snRNA biogenesis phosphodiesterase 1	HGNC:25792	16q21
498	USP18	ubiquitin specific peptidase 18	HGNC:12616	22q11.2
499	USP43	ubiquitin specific peptidase 43	HGNC:20072	17p13.1
500	VAV1	vav guanine nucleotide exchange factor 1	HGNC:12657	19p13.3
501	VPS13B	vacuolar protein sorting 13 homolog B	HGNC:2183	8q22.2

502	VPS45	vacuolar protein sorting 45 homolog	HGNC:14579	1q21.2
503	WAS	WASP actin nucleation promoting factor	HGNC:12731	Xp11.23
504	WDR1	WD repeat domain 1	HGNC:12754	4p16.1
505	WDR44	WD repeat domain 44	HGNC:30512	Xq24
506	WIPF1	WAS/WASL interacting protein family member 1	HGNC:12736	2q31.1
507	WNT2B	Wnt family member 2B	HGNC:12781	1p13.2
508	WNT6	Wnt family member 6	HGNC:12785	2q35
509	WRAP53	WD repeat containing antisense to TP53	HGNC:25522	17p13.1
510	XIAP	X-linked inhibitor of apoptosis	HGNC:592	Xq25
511	XRCC4	X-ray repair cross complementing 4	HGNC:12831	5q14.2
512	ZAP70	zeta chain of T cell receptor associated protein kinase 70	HGNC:12858	2q11.2
513	ZBTB24	zinc finger and BTB domain containing 24	HGNC:21143	6q21
514	ZNF341	zinc finger protein 341	HGNC:15992	20q11.22
